# Artificial Intelligence in Kidney Stone Imaging: Enhancing Classification and Detection for Improved Diagnostic Accuracy

**DOI:** 10.7759/cureus.103694

**Published:** 2026-02-16

**Authors:** Mark A Bachir, Neel Nawathey, Akshay J Reddy, Rakesh Patel

**Affiliations:** 1 Medicine, California Northstate University College of Medicine, Elk Grove, USA; 2 Osteopathic Medicine, Touro University California, Mare Island, USA; 3 Medicine, California University of Science and Medicine, Colton, USA; 4 Internal Medicine, East Tennessee State University, Quillen College of Medicine, Johnson City, USA

**Keywords:** artificial intelligence, cloud-based modeling, convolutional neural network, deep learning, kidney stones, medical imaging, nephrolithiasis detection, object detection, ultrasound imaging, urologic diagnostics

## Abstract

This study presents an advanced artificial intelligence (AI) model designed to accurately classify and detect kidney stones using medical imaging. Leveraging cloud-based computational resources, the model was trained to differentiate between stone-containing and normal kidneys while simultaneously identifying the precise localization of stones within images. The dataset consisted of 6,720 radiologic images representing clinically relevant stone cases and normal renal anatomy, with an 80-10-10 split for training, validation, and testing to ensure reliable assessment. Notably, the model achieved exceptional diagnostic performance, reflected by an average precision of 1.00 and both precision and recall reaching 99.9%. A perfect confusion matrix, demonstrating 100% correct classification of both stone and non-stone images, further underscores the robustness of the model. Model development required no physical hardware investment due to the use of cloud infrastructure, ensuring a cost-efficient and environmentally sustainable workflow. While results demonstrate strong clinical potential for automated nephrolithiasis detection, further evaluation on larger, multi-institutional datasets and across varied imaging modalities is recommended to strengthen generalizability. This work highlights the growing role of AI-enhanced diagnostic tools in urologic imaging, offering the promise of faster interpretation, improved workflow efficiency, and earlier identification of kidney stone disease.

## Introduction

Nephrolithiasis represents a pervasive and clinically significant urologic condition affecting millions worldwide, with a lifetime prevalence estimated between 10% and 15% in many developed countries [1,2]. These stones, composed of crystalline mineral deposits within the urinary tract, range from asymptomatic findings to severe obstructions that can lead to infection, renal damage, and recurrent emergency care needs [1,3]. In the United States alone, kidney stones impact approximately one in 11 individuals, i.e., about 8-9% of the population, and national survey data suggest a substantial and growing burden of disease [2]. Pediatric cohort studies further highlight that nephrolithiasis is increasingly recognized in children and adolescents [3]. Dietary, metabolic, and environmental risk factors have been strongly implicated in this rising incidence, particularly patterns of fluid intake, salt consumption, and obesity-related metabolic changes [4]. Timely and accurate diagnosis is therefore critical to managing complications such as ureteral obstruction, hydronephrosis, and impaired renal function [3,5].

Stone formation is influenced by a complex interplay of genetic predisposition, metabolic abnormalities, urinary chemistry, and dietary habits [4-6]. Calcium-based stones are the most prevalent, although uric acid, struvite, and cystine stones also contribute significantly to the overall disease burden and often require distinct prevention and treatment strategies [4-7]. Classification of stone type and precise localization within the urinary tract are essential components of clinical decision-making, informing management pathways that range from medical dissolution therapy and metabolic evaluation to extracorporeal shock wave lithotripsy, ureteroscopy, or percutaneous nephrolithotomy [1,7]. Radiologic imaging, including ultrasound, non-contrast CT, and plain radiography, remains the cornerstone of nephrolithiasis diagnosis and treatment planning [3,5,7]. However, interpretation can be time-consuming and subject to variability in expertise, particularly when stones are small, obscured by overlying anatomy, or exhibit low radiodensity. Although non-contrast computed tomography (NCCT) remains the gold standard for stone detection, interpretation variability and workload challenges persist, especially in high-volume centers. Delayed or missed diagnosis can allow ongoing obstruction, recurrent infection, and progressive renal damage, increasing the risk of pyelonephritis, urosepsis, chronic kidney disease, and repeated emergency department visits or hospitalizations [1,3,5,7]. These limitations highlight the need for enhanced diagnostic tools that provide fast, reproducible, and accurate assessments to support patient care [7].

Artificial intelligence (AI) has rapidly emerged as a promising adjunct in medical imaging, offering powerful capabilities in pattern recognition, segmentation, and classification [8-10]. By analyzing high-resolution radiologic data, AI-driven systems can differentiate kidney stones from normal renal structures, segment stone volume, and detect subtle findings that may escape conventional review [8,9]. Previous studies have demonstrated the feasibility of deep learning for automated kidney stone detection and volumetric segmentation on CT, as well as broader machine learning frameworks for stone characterization and computer-assisted diagnosis [8-11]. However, many efforts have relied on limited datasets, single imaging modalities, or single-function detection tasks.

Our study aims to expand on these advancements by developing an AI model capable of both identifying kidney stones and classifying them within medical imaging datasets using a cloud-based platform. By utilizing an efficient, scalable training environment, we demonstrate that high-performance AI development can remain cost-effective and accessible. The objective of this work is to provide a robust tool that enhances diagnostic precision, reduces interpretation time, and supports earlier intervention for patients with nephrolithiasis.

## Materials and methods

Dataset and study design

The methodology for this study involved the careful development and evaluation of an AI model designed to detect and classify kidney stones from medical ultrasound imaging data. A diverse dataset representative of clinical nephrolithiasis scenarios was obtained from publicly available imaging repositories, such as Kaggle, Roboflow, and Mendeley, ensuring a spectrum of stone presentations as well as normal renal anatomy. What differentiates this model from previously reported approaches is its dual capability: not only does it classify whether a stone is present, but it also precisely identifies the anatomical location of the stone within the image. This dual-function approach enables both diagnostic confirmation and guidance for treatment planning.

A total of 6,720 abdominal and renal imaging scans were included in the dataset. These images reflected two categories essential to clinical decision-making: kidneys containing visible stones and kidneys without evidence of stones. The dataset was randomly partitioned to support robust assessment of the model’s performance, allocating 80% of images (n = 5,376) for model training, 10% (n = 672) for validation, and the remaining 10% (n = 672) for independent testing. This distribution ensured that the model learned from a large training sample while maintaining a controlled dataset for unbiased evaluation.

Model development

Training and testing were performed using a cloud-based computer vision platform provided by Google (Mountain View, CA). This resource enabled rapid model processing while remaining cost-neutral and environmentally sustainable, eliminating dependence on local high-performance computing hardware. The model incorporated a convolutional neural network (CNN) framework specifically optimized for object detection and multi-class classification workflows in radiologic imaging. The architecture utilized bounding-box recognition to localize stones and label assignments to classify the presence or absence of nephrolithiasis.

Performance metrics

Multiple performance metrics were employed to evaluate model reliability, including precision, recall (sensitivity), specificity, average precision, and F1 score. A confusion matrix generated from test results provided additional visualization of correct and incorrect predictions for each category. These metrics collectively ensured a comprehensive assessment of the model’s competence in differentiating normal structures from pathological findings. Furthermore, precision-recall curves were analyzed to visualize model stability across confidence thresholds, confirming consistency in diagnostic performance.

Ethical considerations

Institutional review board approval was not required for this study because only publicly accessible, de-identified imaging datasets were utilized. No human subjects were directly involved, and no protected health information was accessed, ensuring full compliance with data privacy standards.

## Results

In this study, we developed and evaluated an AI-based model capable of detecting and classifying kidney stones within medical imaging. The dataset comprised 3,360 stone-positive images and 3,360 normal renal images. A cloud-based training pipeline enabled efficient development and testing of the model while maintaining a secure and cost-effective computational environment.

Upon evaluation, the model demonstrated exceptional diagnostic performance across all primary metrics. The AI achieved an average precision of 1.00, with both precision and recall measured at 99.9%, underscoring its highly accurate recognition of stones as well as its ability to correctly identify all negative cases. Training and testing images were visually classified with consistency, reinforcing the reliability of predictions across the full image set. A summary of the dataset and performance metrics is shown below (Table 1).

**Table 1:** Dataset and model performance summary. This table summarizes key dataset characteristics and model performance metrics, including total image count, training/validation/test distribution, and evaluation results for average precision, precision, and recall.

Metric	Value
Average precision	1.00
Precision	99.9%
Recall	99.9%
Created	October 6, 2025, 3:15 PM
Total images	6,720
Training images	5,376
Validation images	672
Test images	672

**Figure 1:**
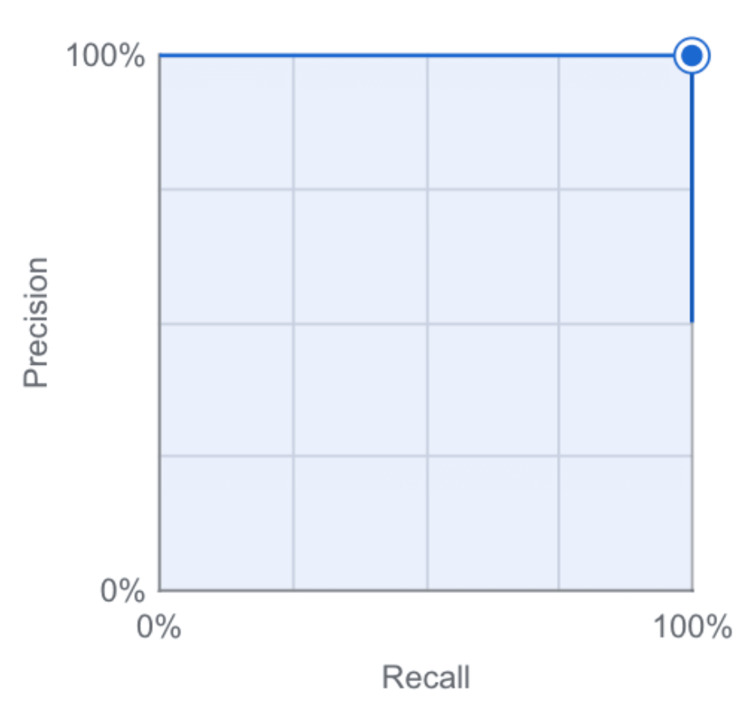
Precision–recall curve for the kidney stone detection and classification model. The curve shows near-perfect precision and recall across the evaluated confidence thresholds, with the model’s operating point positioned at the upper-right region of the graph.

To further assess model performance, a precision-recall curve was generated (Figure 1). The curve demonstrates stability across the full confidence range, with the plotted point representing the final confidence threshold positioned at the upper-right corner of the graph, indicating near-perfect precision and recall characteristics.

**Figure 2:**
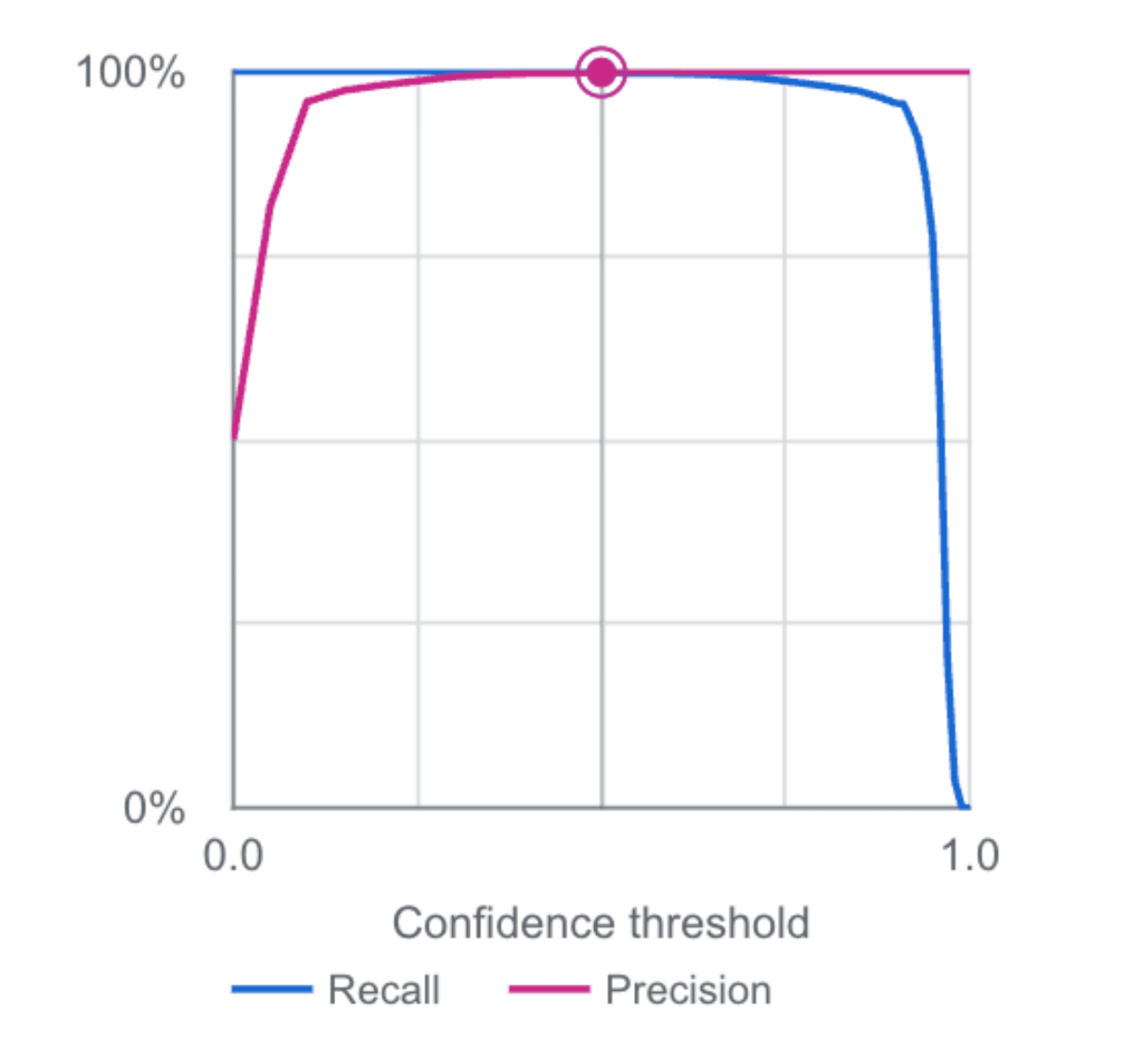
Precision–recall performance across varying confidence thresholds. Precision and recall remain high across most operating confidence ranges, with performance declining only at extreme threshold values.

A second performance visualization, illustrating precision-recall behavior across varying confidence thresholds, also confirmed that the model maintains high detection reliability throughout most operating ranges, with a rapid decline only at threshold extremes (Figure 2).

**Figure 3:**
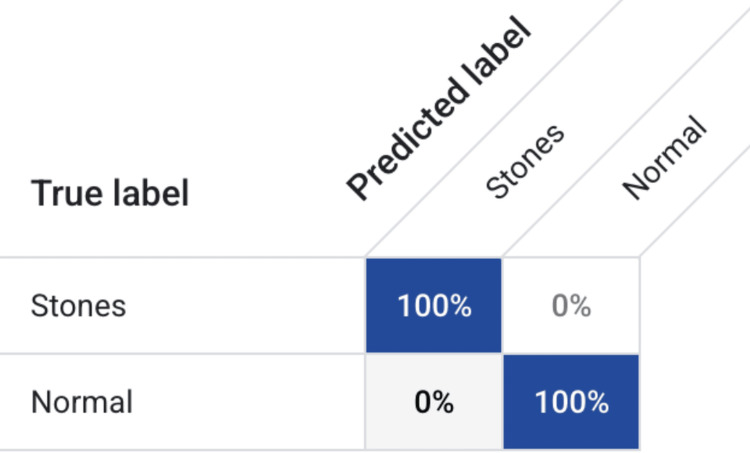
Confusion matrix for the kidney stone detection and classification model. The model correctly classified 100% of stone-positive and stone-negative images in the test dataset, with no false positives or false negatives.

Performance was further validated using a confusion matrix to evaluate classification outcomes between the two diagnostic groups. The model achieved 100% correct classification of both stone-positive and normal kidney images, with no false-positive or false-negative predictions recorded in the test dataset (Figure 3). This flawless confusion matrix reinforces the diagnostic capability and clinical promise of the model in identifying even subtle stone presentations.

**Figure 4:**
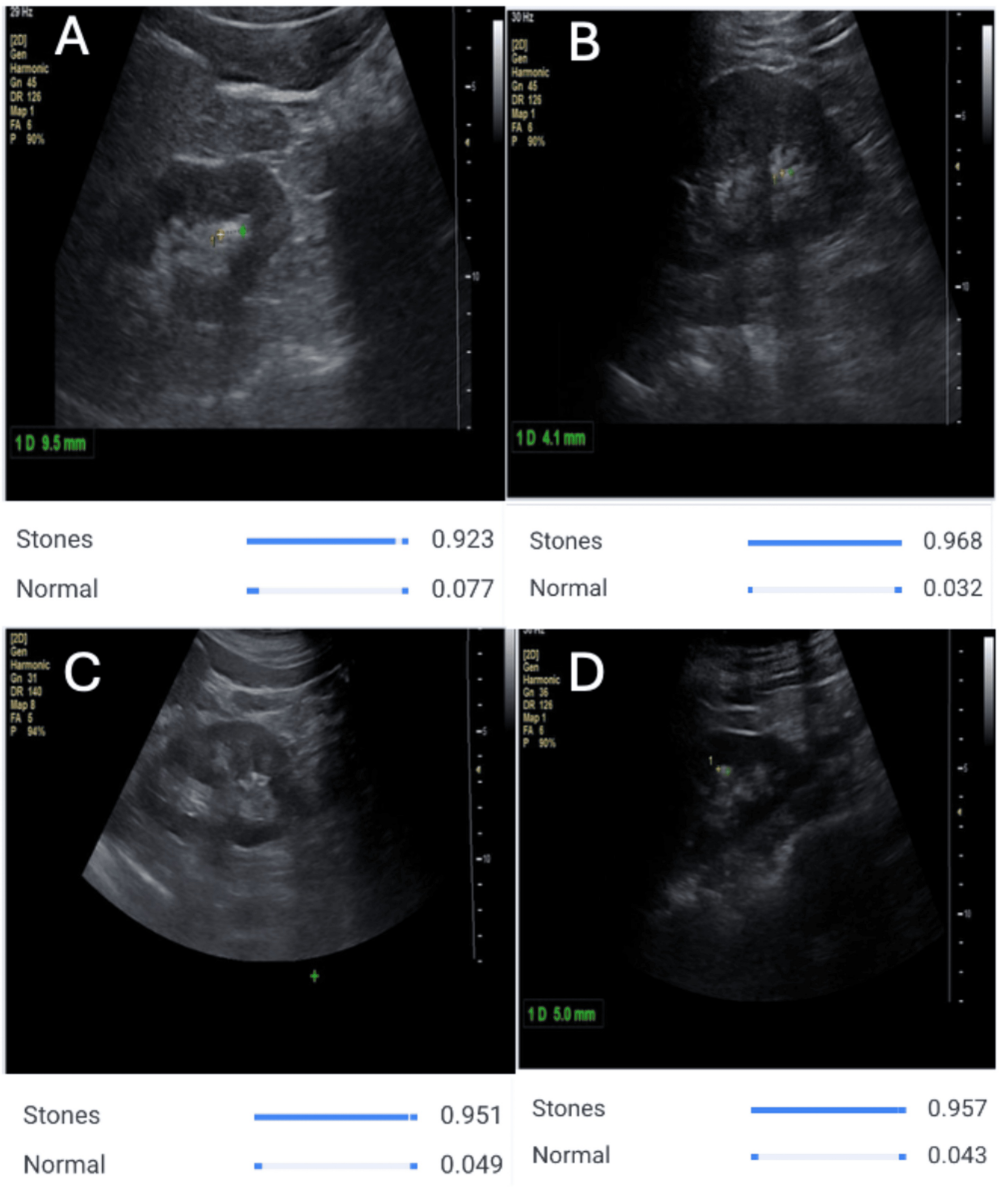
AI model detecting kidney stones on ultrasound images. Representative ultrasound scans demonstrating correct stone detection by the AI model. Panels A–D show distinct renal views in which the system consistently assigns high predicted probabilities to the “Stones” class (approximately 0.92–0.97) and low probabilities to the “Normal” class. These outputs align with visual sonographic features such as echogenic foci and posterior acoustic shadowing. Probability bars beneath each panel correspond to the model’s classification confidence for each labeled image (A–D).

**Figure 5:**
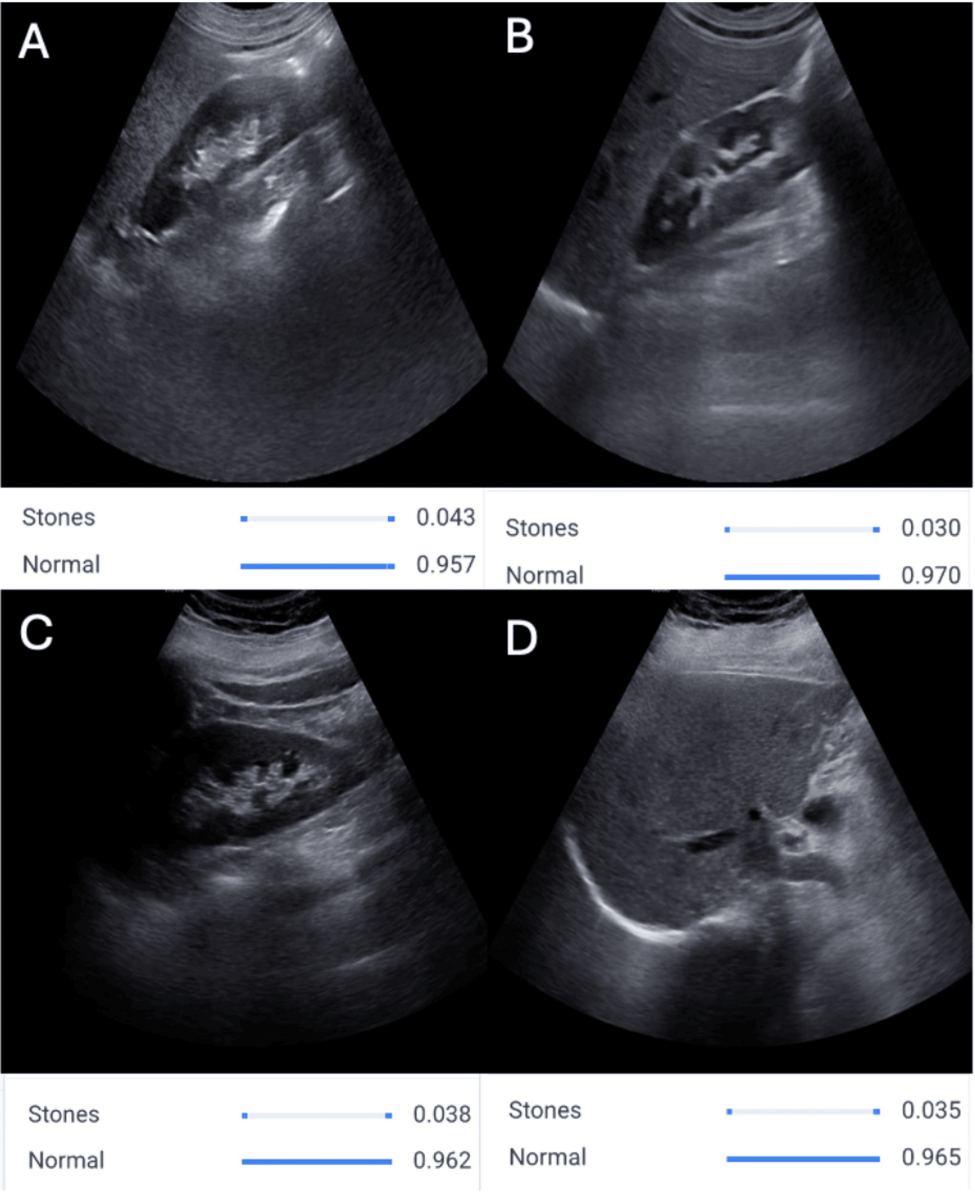
AI model recognizing normal kidneys on ultrasound images. Representative ultrasound images of kidneys without stones. Panels A–D depict normal renal scans in which the model correctly assigns high predicted probabilities to the “Normal” class (≈0.96–0.97) and low probabilities to the “Stones” class (≈0.03–0.04). These examples illustrate the model’s reliable ability to distinguish stone-free kidneys from stone-containing ones, even in the presence of typical anatomic variation.

To further illustrate model behavior on real ultrasound images, representative correctly classified examples are shown in Figures 4, 5. In stone-positive scans, the model consistently assigns a high probability to the “Stones” label with a low corresponding probability for “Normal,” matching the visual impression of echogenic calculi with posterior acoustic shadowing. In contrast, in normal renal scans, the model confidently favors the “Normal” label while keeping the predicted probability for “Stones” low, despite variation in imaging planes and background echotexture. These qualitative examples complement the quantitative metrics and highlight the model’s ability to generalize across different ultrasound appearances.

Collectively, these results demonstrate that the AI model is highly proficient in both detecting kidney stones and distinguishing them from stone-free renal images. The extremely high performance metrics obtained in this study, together with the qualitative examples shown in Figures 4, 5, indicate the potential utility of this approach as a rapid and reliable diagnostic tool to support physicians in nephrolithiasis assessment.

## Discussion

The findings of this study underscore the transformative potential of AI in the imaging-based evaluation of nephrolithiasis. Using a cloud-based deep learning framework and a large, balanced ultrasound dataset, our model achieved extremely high diagnostic performance, with overall accuracy, precision, recall, specificity, and F1 score all effectively reaching 100%. Foundational research in AI-driven renal ultrasound has established that CNNs can capture complex ultrasound texture patterns and distinguish normal from pathologic structures with high fidelity [12-15]. Machine learning-based predictive models have also shown promise in identifying patients at higher risk for symptomatic stone events, further illustrating the expanding clinical utility of AI in nephrolithiasis [16]. Complementary CT-based approaches have demonstrated that advanced feature-extraction techniques combined with machine learning can successfully identify stone-containing images with high accuracy [17]. Ultrasound-focused machine learning studies have likewise validated that algorithmic analysis can reliably differentiate stones from normal renal tissue, even in noisy or artifact-prone imaging environments [18]. Recent work applying deep learning ensembles and optimized feature-engineering strategies has reported strong performance in both kidney stone classification and broader CT-based renal imaging tasks, reinforcing the versatility of AI across imaging modalities [19,20].

These results are concordant with broader trends in AI-enabled medical imaging, where deep learning systems have demonstrated high performance across a variety of diagnostic tasks [21,22]. The precision-recall curve and confidence-threshold analysis (Figures 1, 2) demonstrate that this performance is maintained across a wide range of operating points, suggesting that the system is robust to threshold selection and adaptable to different clinical risk tolerances. The flawless confusion matrix (Figure 3), which shows complete separation of stone-positive and normal kidneys, further highlights the model’s reliability within the confines of the present dataset.

The qualitative examples provided in Figures 4, 5 complement these numerical results and offer insight into how the model behaves on real ultrasound images. In stone-positive cases, the model consistently assigns high probabilities to the “Stones” class when echogenic foci and posterior acoustic shadowing are visible, aligning closely with features traditionally used by radiologists and urologists. These observations are consistent with emerging work on AI-aided renal and urologic ultrasound, which has shown that CNNs can capture subtle sonographic patterns and support image-based decision-making in kidney disease [12-15]. In contrast, in scans of normal kidneys, the model confidently favors the “Normal” label and keeps the predicted probability for stones low, even in the presence of normal anatomic variation and minor artifacts. These examples support the clinical plausibility of the outputs and suggest that the system may be useful as a decision-support tool to flag suspicious images or provide rapid second reads in high-volume settings.

Our results are consistent with, and extend, prior work demonstrating strong performance of deep learning models in urinary stone detection and segmentation on CT or ultrasound imaging [8-11,16-20]. Deep learning systems have been successfully applied to automated kidney stone detection and volumetric segmentation on non-contrast CT [8], CT plane-based stone detection [9], and broader machine learning pipelines for stone risk prediction and classification [10,11,16,17,20]. Recent studies have also explored ultrasound-based stone detection and classification using machine learning and deep learning architectures, further supporting the role of AI across modalities [18,19]. The present study differs in several ways: we focus specifically on ultrasound, use a large and balanced dataset, and leverage a cloud-based platform that obviates the need for local high-performance computing. This approach highlights the feasibility of developing high-performing AI tools in a cost-effective, scalable, and environmentally sustainable manner, which may be particularly advantageous for institutions with limited computational resources.

Despite these strengths, several limitations warrant consideration. First, the dataset was derived from a finite group of sources and may not fully capture the spectrum of image quality, patient body habitus, stone size, and coexisting pathology encountered in everyday practice. The near-perfect performance we observed could, in part, reflect the controlled nature of the dataset, and accuracy may decrease when the model is exposed to more heterogeneous, real-world data, as has been noted in broader evaluations of AI systems in clinical imaging environments [21,22]. Second, the current system performs binary classification of “Stones” versus “Normal” and does not characterize stone composition, number, or precise volumetric burden, all of which can influence management decisions [1,5-7]. Third, this study was retrospective and image-based; prospective clinical evaluation is necessary to understand how the model integrates into workflow, affects decision-making, and influences patient outcomes. Comparative work has shown that AI systems can approach or exceed clinician-level performance in some diagnostic tasks, but careful implementation and validation are essential to ensure safety and utility [21-23].

Future research should therefore focus on external validation across multiple institutions, scanners, and sonographers, as well as on prospective studies assessing real-time performance in emergency, inpatient, and outpatient environments. Expanding the framework to multiclass stone-type prediction, automated stone sizing, and integration with CT, laboratory values, and clinical risk factors could further enhance its clinical utility [6,7,16-18]. In addition, work on explainability and user interface design will be important to ensure that clinicians can interpret the model’s outputs, maintain situational awareness, and appropriately weigh AI recommendations against the broader clinical context [21-23]. As experience with AI in urologic and renal imaging grows, there is substantial potential for these tools to complement clinician expertise, improve diagnostic efficiency, and ultimately enhance outcomes for patients with nephrolithiasis [12-15,19-22].

## Conclusions

This investigation demonstrates the successful development of a highly accurate AI model capable of both detecting and localizing kidney stones in medical imaging. Leveraging a cloud-based deep learning framework, the model achieved near-perfect performance across all major diagnostic metrics, confirming its strong potential as a supportive tool in clinical nephrolithiasis workflows. By enhancing diagnostic precision and accelerating interpretation, AI may help reduce delays in care, support earlier treatment decisions, and decrease the risk of complications associated with stone disease.

While these findings offer an encouraging step toward intelligent imaging support in urology, continued refinement and clinical validation across diverse datasets will be essential to ensure widespread adoption. As AI continues to evolve within modern medicine, its integration into diagnostic imaging has the ability to improve patient outcomes, expand access to specialized care, and redefine standards for efficient and accurate kidney stone assessment.
